# Supplementary material for: Spotting of Volatile Signatures through GC-MS Analysis of Bacterial and Fungal Infections in Stored Potatoes (Solanum tuberosum L.)
Source: Foods. 2023 May 22;12(10):2083. doi: 10.3390/foods12102083 (PMC10216920; doi:10.3390/foods12102083)
Supplement: Supplementary file 1 [file foods-12-02083-s001.zip › foods-2297468-supplementary.pdf]

Table S1. VOCs of control and infected potatoes stored under selected storage conditions (*note: The values are means of 3 replications)

			Area percentage of VOCs											
**VOCs**	**class**		**Control sample**			***Pectobacterium carotovorum* (PB) infected**			***Aspergillus niger* (AN) infected**			***Aspergillus flavus* (AF) infected**		
**T=25 °C**			**W1**	**W2**	**W3**	**W1**	**W2**	**W3**	**W1**	**W2**	**W3**	**W1**	**W2**	**W3**
2-(Methylthio)nicotinic acid	acid	AC1	-	-	-	-	-	-	-	-	-	5.67±1.34	5.67±2.08	5.67±1.23
Bicyclo[2.2.1]heptane-2-carboxylic acid, 2-propyl-	acid	AC2	-	-	-	-	-	4.34±1.55	-	-	-	-	-	-
Acetic acid	acid	AC3	-	-	-	-	-	-	-	-	-	0.33±0.06	0.33±0.02	0.33±0.45
Butanedioic acid	acid	AC4	12.73±1.06	-	-	-	-	-	-	-	-	-	-	-
Propanoic acid, 3-hydroxy-	acid	AC5	-	3.58±0.56	4.12±1.1	-	4.69±1.06	14.21±1.96	-	-	3.83±0.03	-	-	-
2-Oxopropionic acid	acid	AC6	2.9±0.03	-	-	-	-	-	-	-	-	-	-	-
Benzenepropanoic acid, 3-methoxy-.alpha.,4-bis[(trimethylsilyl)oxy]-,	acid	AC7	-	-	-	-	-	-	-	-	-	-	-	-
Benzoic acid, 2,5-dichloro-3,6-dimethoxy-,	acid	AC8	-	-	-	1.66±0.06	-	-	-	-	-	-	-	-
butanedioic acid, 2-cyano-2,3-dimethyl-	acid	AC9	-	1.51±0.09	4.46±1.58	8.32±2.14	2.3±0.08	1.4±0.75	8.86±2.03	4.2±0.60	1.31±0.69	5.01±1.58	5.01±2.15	5.01±1.58
Carbamic acid, (3-chlorophenyl)-	acid	AC10	-	-	-	-	0.2±	-	-	-	-	-	-	-
carbamic acid, N-1-naphthalenyl-, 4-(diethylamino)-2-	acid	AC11	-	-	-	5.9±1.89	-	-	-	-	-	-	-	-
carbonic acid,	acid	AC12	-	7.25±2.98	-	-	-	-	-	-	-	-	-	-
Carbonic acid, monoamide, N-isobutyl-	acid	AC13	-	-	-	-	-	-	4.56±1.23	-	-	-	-	-
CH3CHClCN	acid	AC14	-	-	-	-	39.96±3.74	-	-	7.08±2.87	2.6±0.58	-	-	-
Fumaric acid	acid	AC15	-	-	-	-	-	-	-	-	-	-	-	-
Glutaric acid	acid	AC16	-	-	-	-	-	-	-	-	4.73±1.23	-	-	-
Acetic acid, 2-methyl-2-phenyl-	acid	AC17	-	-	-	-	3.77±0.51	-	-	-	-	-	-	-
Bicyclo[3.1.1]hept-2-ene-2-ethanol, 6,6-dimethyl-	alcohol	AL1	-	-	8.56±2.12	-	-	11.34±2.42	-	-	-	-	-	-
1H-pyrrole-3-carboxaldehyde, 2,5-dimethyl-1-phenyl-	aldehyde	ALD1	-	-	-	-	-	-	-	15.19±2.06	-	-	-	-
5,6-Dihydro-2,3,9,11-tetrachlorobenz[c]acridine-7-carboxal dehyde	aldehyde	ALD2	-	-	-	-	-	-	-	1.75±0.87	-	-	-	-
9,12-Octadecadienal, dimethyl acetal	aldehyde	ALD3	-	-	8.35±1.73	-	-	-	-	-	-	-	-	-
Propanal, 2-methyl-, (2,4-dinitrophenyl)hydrazone	aldehyde	ALD4	-	-	-	-	-	1.32±0.14	-	-	-	-	-	-
1,4-Dioxane	alkane	ALK1	-	-	-	-	6.03±1.42	-	4.21±1.74	-	-	-	-	-
1-Chloromethyl-1-(2-butoxy)-1-silacyclohexane	alkane	ALK2	-	-	-	-	-	-	-	-	-	0.26±0.06	-	-
1-Dimethyl(3-chloropropyl)silyloxydecane	alkane	ALK3	-	3.7±0.95	-	-	-	-	-	-	-	-	-	-
3,5-Dimethyl-1-dimethylsilyloxycyclohexane	alkane	ALK4	-	-	-	-	-	-	-	-	-	-	2.18±0.58	-
Bicyclo[2.2.2]octane, 1-chloro-	alkane	ALK5	-	-	-	-	-	-	5.99±1.35	-	-	-	-	-
Ethane	alkane	ALK6	-	-	-	-	-	-	-	-	-	-	-	2.11±1.57
Phenol, 4-(aminomethyl)-2-methoxy-	alkane	ALK7	-	-	-	-	-	-	-	-	-	-	-	1.57±0.09

1,2-Bis(4,6,8-trimethyl-1-azulenyl)ethylene	alkene	ALE1	-	-	-	-	-	-	30.33±3.23	-	-	-	-	-
1-Propene, 3-azido-	alkene	ALE2	1.45±1.02	-	-	-	-	-	-	-	-	-	-	-
Ethylene	alkene	ALE3	-	3.01±1.08	-	3.71±0.23	1.21±0.28	-	-	-	-	-	-	0.41±0.01
9-Octadecenamide, N-(1-methylethyl)-	amide	AM1	-	-	2.22±0.08	-	-	-	-	-	-	-	-	-
Acetamide, 2-(2-thiophenyl)-N-ethyl-N-nonyl-	amide	AM2	-	-	-	-	-	2±0.03	-	-	-	-	-	-
Acetamide, N-[4-(acetylamino)butyl]-N-[3-(acetylpentylamino)propyl]-	amide	AM3	-	-	-	-	-	-	-	-	-	-	-	1.4±0.09
acetamide, N-[4-[[1,5-dihydro-3-methyl-5-oxo-1-phenyl-4H-pyrazol-4-ylidene]amino]phenyl]-N-methyl-	amide	AM4	-	-	-	-	-	-	4.76±1.58	-	-	-	-	-
hexanamide, 2-[2,4-bis(1,1-dimethylpropyl)phenoxy]-N-(2,3-dihydro-2-oxo-6-benzoxazolyl)-	amide	AM5	-	-	-	8.72±2.93	-	-	-	-	-	-	-	-
2,5,6-Tribromopyridin-3-ylamine	amine	AMN1	-	-	-	-	-	-	-	-	3.89±1.05	-	-	-
Aposcopolamine	amine	AMN2	-	-	-	-	-	-	-	-	1.05±0.08	-	-	-
1,2-Dimethyl-3-(1',1',2'-trichloro-3-ethylallyl)benzene	aromatic	AR1	-	-	-	-	-	-	-	-	-	-	2.42±0.02	-
2-[2-Amino-5-(4-chlorophenyl)pyrimidin-4-yl]-5- [illegible]	aromatic	AR2	2.16±1.23	-	-	-	-	-	-	-	-	-	-	-
Benzene, 1,2,4-trichloro-5-nitro-	aromatic	AR3	-	-	-	9.25±2.07	-	-	-	-	-	-	-	-
Benzene, 1-methoxy-2,4-dinitro-	aromatic	AR4	-	-	-	-	-	-	-	-	-	6.42±1.81	-	-
Phenyltrichlorogermane	aromatic	AR5	1.61±0.56	-	-	-	-	-	-	-	-	-	-	-
benzenamine, 4,4',4''-methylidynetris[2-methyl-	aromatic	AR6	-	-	-	-	-	-	-	-	-	0.71±0.01	-	-
Benzenamine, 4,4',4''-methylidynetris[N,N-dimethyl-	aromatic	AR7	7.89±1.71	23.42±2.54	-	-	0.39±0.05	-	0.96±0.07	-	-	1.21±0.41	33.82±3.69	1.47±0.05
benzenamine, 4-[bis(2,4,6-trimethylphenyl)boryl]-N,N-dimethyl-	aromatic	AR8	-	-	3.59±0.14	-	-	-	-	-	-	-	-	-
Butyric acid hydrazide	azide	AZ1	-	1.26±0.06	-	-	-	-	-	-	-	-	-	-
Carbohydrazide	azide	AZ2	-	-	-	-	-	-	-	-	9.79±2.43	-	-	-
Methanethione, bis(4-methoxyphenyl)-	azide	AZ3	-	2.99±0.31	-	-	-	-	-	-	-	-	-	-
11H-Dibenzo[b,e][1,4]dioxepin-7-carboxylic acid, 3,8-dimethoxy-1,4,6,9-tetramethyl-11-oxo-, methyl ester	ester	EST1	-	-	-	-	-	-	-	-	-	12.18±2.87	-	-
2-Benzylthio-3-(2-oxocyclohexyl)propionic acid, t-butyl ester	ester	EST2	-	-	-	-	-	-	0.7±0.02	-	-	-	-	-
2-Hydroxyethyl hydrogen vinylphosphonate	ester	EST3	-	-	-	-	-	-	-	-	-	-	-	1.86±0.08
3,3-Dimethoxy-4-nitrobutyric acid, methyl ester	ester	EST4	-	-	-	-	-	-	-	-	-	-	0.62±0.02	-
4-(3,4-Dichlorobenzylidene)-1-isobutyl-2-methyl-5-oxo-4,5-dihydro-1H-pyrrole-3-carboxylic acid, methyl ester	ester	EST5	-	-	-	-	-	-	-	-	-	3.02±1.89	-	-
6-Aminocaproic acid, n-butoxycarbonyl-	ester	EST6	-	-	-	-	-	-	-	-	-	1.69±0.74	-	-

, heptyl ester														
7H-[1,3,4]Thiadiazolo[3,2-a]pyrimidine-5-carboxylic acid, 2-(methylthio)-7-oxo-, ethyl ester	ester	EST7	-	-	-	-	-	-	5.68±1.36	-	-	-	-	-
Dimethyl 1,2-di(2-methyl-1-propenyl)tricyclo[3.1.0.0(2,4)]hexane-3,6-dicarboxylate	ester	EST8	-	-	-	1.94±0.05	-	-	-	-	-	-	-	-
Ethyl 4-amino-2-[2,4-dichlorobenzylthio]-5-pyrimidine carboxylate	ester	EST9	-	1.63±0.04	-	-	-	-	-	-	-	-	-	-
Ethyl 5-amino-1-(4,7-dimethyl-1,3-benzothiazol-2-yl)pyrazole-4-carboxylate	ester	EST10	-	-	-	-	-	-	-	-	-	1.86±0.28	-	-
Ethyl formate	ester	EST11	-	-	-	-	0.46±0.42	-	-	7.88±2.13	-	-	-	-
Ethyl N,N-diphenylcarbamate	ester	EST12	-	-	4.31±1.36	-	-	-	-	-	-	-	-	-
Glycine, N,N-bis(2-ethoxy-2-oxoethyl)-, ethyl ester	ester	EST13	-	-	1.02±0.06	-	0.81±0.10	3.16±1.53	-	-	-	2.93±1.05	3.98±1.09	-
Hydrazinecarboxylic acid, ethyl ester	ester	EST14	-	-	-	-	-	-	-	2.74±1.74	-	-	-	-
Isonicotinic acid, phenylmethyl ester	ester	EST15	-	-	-	-	-	2.67±0.85	-	-	-	-	-	-
Lycoramine	ester	EST16	-	-	-	-	-	-	-	-	-	-	-	1.5±0.50
N-Chlorosuccinimide	ester	EST17	-	1.55±0.06	-	-	-	-	-	-	-	-	-	-
propanoic acid, 3-chloro-, 2-chloroethyl ester	ester	EST18	-	-	2.09±0.45	-	-	-	-	-	-	-	-	-
Propylene Carbonate	ester	EST19	-	2.49±1.12	-	-	-	-	-	-	-	-	-	-
Succinic acid, ethyl 2-methoxy-4-chlorobenzyl ester	ester	EST20	-	-	-	-	1.01±0.21	-	-	-	-	-	-	-
Succinic acid, phenyl 4-isopropylphenyl ester	ester	EST21	-	-	-	-	-	-	-	-	-	-	-	1.4±0.89
Terephthalic acid, butyl isopropyl ester	ester	EST22	-	-	-	-	-	-	-	-	-	-	2.42±0.74	-
Thiocyanic acid, methyl ester	ester	EST23	-	-	13.95±2.13	-	-	-	-	-	-	-	-	-
1,2-Oxathiolane, 2,2-dioxide	ether	ETH1	-	-	-	-	-	17.8±1.69	-	-	-	-	-	-
2,3,4,5,6-Pentabromobenzyl alcohol, n-butyl ether	ether	ETH2	-	-	-	-	5.59±1.05	-	-	-	-	-	-	-
2-Azido-3-ethoxy-pyridine 1-oxide	ether	ETH3	-	-	0.55±0.02	-	-	-	-	-	-	-	-	-
3-Pyridazinamine 2-oxide	ether	ETH4	-	-	-	-	-	-	-	-	-	-	-	1.96±0.01
Cyacetacide	ether	ETH5	-	-	-	-	-	-	-	-	2.73±0.08	-	-	-
Prothioconazole, N-methyl-, methyl ether	ether	ETH6	-	-	-	-	-	-	-	6.94±1.28	-	-	-	-
Tetraphenylphthalic anhydride	ether	ETH7	-	-	-	10.4±1.39	-	-	-	-	-	-	-	-
Trimethylene oxide	ether	ETH8	-	4.8±1.45	-	-	-	-	-	-	-	-	-	-
1,3-Cyclopentanedione	ketone	KET1	-	-	-	-	-	-	-	-	-	-	-	4.97±
1,4-Cyclohexanedione	ketone	KET2	-	-	-	-	-	-	-	-	-	-	2.88±0.78	-
2(1H)-Pyridinone, 1-(2-methylphenyl)-	ketone	KET3	-	-	2.63±0.26	-	-	-	-	-	-	-	-	-
2,6-Bis(4-azidobenzylidene)-4-methylcyclohexanone	ketone	KET4	3.22±0.78	-	-	2.01±0.42	-	-	1.45±0.12	-	-	-	8.78±1.36	-
4H-Thiopyran-4-one, tetrahydro-	ketone	KET5	1.81±0.04	-	-	-	-	-	-	-	-	-	-	-
4-Methyl-3,4-dihydro-[1,2,3]triazolo[4,5-d]pyrimidine-5,7-dione	ketone	KET6	-	-	4.16±0.85	-	-	-	-	-	-	-	-	-

7-Hydroxy-3,5,7,8-tetrahydro-4,6-pteridinedione	ketone	KET7	-	-	-	-	-	-	-	-	-	-	3.96±0.25	-
7-Oxabicyclo[4.2.1]nona-2,4-dien-8-one	ketone	KET8	-	-	-	7.69±1.25	-	-	-	-	-	-	-	-
Bis-(3-azido-4-methoxy-phenyl)methanone	ketone	KET9	-	-	1.76±0.04	-	-	3.55±1.28	-	-	-	-	-	-
Ethanone, 1-[4-(methyltelluro)phenyl]-	ketone	KET10	-	2.33±0.02	-	-	-	-	-	-	-	-	-	-
1,2,4,5-Tetrazine	nitrogen	N1	-	-	-	-	3.64±0.25	-	-	-	-	-	-	-
Acetonitrile, hydroxy-	nitrogen	N2	-	11.83±1.14	-	-	-	-	5.33±0.25	-	-	-	-	-
Cyclohexasiloxane, dodecamethyl-	nitrogen	N3	13.62±1.58	-	-	-	-	-	-	-	-	1.22±0.09	-	-
Hydrazine	nitrogen	N4	-	-	-	-	-	-	-	-	4.18±0.41	33.73±2.36	-	-
Hydrazine, methyl-	nitrogen	N5	-	-	-	-	1.15±0.03	-	-	-	-	-	-	1.28±0.04
Propanedinitrile, (1-methylethylidene)-	nitrogen	N6	-	-	-	-	-	-	-	3.48±1.07	-	-	-	-
Propanenitrile	nitrogen	N7	-	-	2.42±0.05	-	-	-	-	-	-	-	-	-
[1,2-Bis(dicyclohexylphosphino)ethane][2-nickelatricyclo[7.4.1.1(3,8)]pentadeca-1(13),3,5,7,9,11-hexaene] complex	other	OTH1	-	-	-	-	-	-	-	3.78±1.58	-	-	-	-
[2]Benzopyrano[7,6-f]indazole-8-acetic acid, 2,4,6,8,9,11-hexahydro-5,10-dihydroxy-2,6-dimethyl-4,11-dioxo-, methyl ester	other	OTH2	4.66±0.58	-	-	-	-	-	-	-	-	-	-	-
1-(4-Methoxy-3-nitrobenzyl)isoquinoline	other	OTH3	7.32±1.47	-	-	-	-	-	-	-	-	-	-	-
1,1'-Biphenyl, 2,2',3,3',4,5,5',6-Octachloro-	other	OTH4	-	-	-	-	11.06±2.03	-	-	-	-	-	-	-
1,2,3-Propanetriol, 1-(1-phenyl-1H-pyrazolo[3,4-b]quinoxalin-3-yl)-, triacetate (ester), [S-(R*,S*)]-	other	OTH5	-	-	-	-	-	-	1.37±0.07	-	-	-	-	-
1,2-Bis(4-methoxycarbonyl-5-phenyl-2-thiazolyl)hydrazine	other	OTH6	-	-	-	-	-	-	-	-	-	0.28±0.01	-	-
1,2-Dimethyl-4-(2-oxo-4-hydroxybutylidene)-trans-decahydroquinoline	other	OTH7	-	-	-	-	-	-	-	-	1.88±0.02	-	-	-
1,3-dioxane-5-methanol, 5,5'-[oxybis(methylene)]bis[2-phenyl-	other	OTH8	-	-	-	-	-	-	-	23.69±2.58	-	-	-	-
1,3-Dioxolo[4,5-b]acridin-10(5H)-one, 6,11-dimethoxy-5-methyl-	other	OTH9	-	-	-	-	-	-	0.41±0.01	-	-	-	-	-
1,3-oxazino[6,5-g][1,3]benzoxazine, 3,8-dicyclohexyl-2,3,4,7,8,9-hexahydro-5,10-bis(1-phenylethyl)-	other	OTH10	-	-	-	-	-	-	-	-	1.4±0.01	-	-	-
1H-Indole, 2-methoxy-3,5-dimethyl-1-(trimethylsilyl)-	other	OTH11	-	-	-	-	-	-	-	-	2.38±1.47	-	-	-
1H-indole, 3,3'-(phenylmethylene)bis[2-phenyl-	other	OTH12	-	-	-	-	-	6.24±1.89	-	-	-	-	-	-
1H-pyrido[2,1-b]benzothiazole-4-carboxaldehyde, 2-[2-(1,3-diethyl-1,3-dihydro-	other	OTH13	-	-	-	-	-	-	-	-	-	-	0.78±0.03	-
1-Naphthacenecarboxylic acid, 2-ethyl-1,2,3,4,6,11-hexahydro-2,4,5,7,10-	other	OTH14	-	-	-	-	-	-	-	-	-	2.94±0.02	-	-

pentahydroxy-6,11-dioxo-, methyl ester														
1-p-[N-[2-Amino-3-cyanopyrazine-5-yl-methyl]-N-methylamino]benzoylaminoadamantane	other	OTH15	-	-	-	-	-	-	-	-	-	1.03±0.01	-	-
2,3,3',4,4',5,5',6-Octachloro-1,1'-biphenyl	other	OTH16	-	-	-	-	-	-	-	-	-	-	-	1.41±1.05
2,4,5-Trihydroxypyrimidine	other	OTH17	-	3.73±1.89	-	-	-	-	-	-	-	-	-	-
2,4-pentadienenitrile, 2-acetyl-5-(dihexylamino)-	other	OTH18	-	-	-	-	4.64±1.04	-	-	-	-	-	-	-
2-Butene ozonide	other	OTH19	-	-	-	-	-	-	-	-	-	-	-	55.53±2.78
2H-Cyclopentathiazol-2-imine, 3,4,5,6-tetrahydro-3-hydroxy-	other	OTH20	-	-	-	-	-	-	-	-	-	3.85±0.45	-	-
2-naphthalenecarboxamide, 1-hydroxy-N-[4-[(octadecylamino)sulfonyl]phenyl]-	other	OTH21	-	-	-	-	-	-	1.98±0.12	-	-	-	-	-
2-Pyrazoline-1-carboxamide, 5-(p-methoxyphenyl)-3-methyl-	other	OTH22	-	-	-	-	4.13±1.54	-	-	-	-	-	-	-
3-(2-Methoxy-phenyl)-2-phenylsulfanylmethyl-3H-quinazolin-4-one	other	OTH23	-	-	-	-	-	-	1.42±0.06	-	-	-	-	-
3-(3,4-(Methylenedioxy)phenyl)-1-(2-quinolyl)-2-propen-1-one	other	OTH24	-	-	-	-	-	-	-	-	-	1.52±0.08	-	-
3-(N,N-Dimethylamino)-4-nitro-9-methylcarbazole	other	OTH25	-	-	-	-	-	-	-	-	-	-	-	1.35±0.74
3-(t-Butyl)-5-azopyrazole	other	OTH26	-	-	3.31±0.14	-	-	-	-	-	-	-	-	-
3H-Naphth[1,8a-b]oxiren-2(1aH)-one, hexahydro-	other	OTH27	-	-	-	-	-	-	-	-	0.59±0.01	-	-	-
3-Methyl-1,2-diazirine	other	OTH28	-	-	-	-	-	-	-	-	-	-	4.45±1.50	-
3-Pyridinecarboxylic acid, 2,5,6-trichloro-4-[[(2,6-dimethyl-4-morpholinyl)thioxomethyl]thio]-, ethyl ester	other	OTH29	-	8.79±1.87	-	-	-	-	-	-	-	-	-	-
4-Acetyl-2-ethoxy-6-methoxyquinoline	other	OTH30	-	-	-	-	-	-	-	-	3.88±1.24	-	-	-
5,6,7,8-Tetrahydroacridine, 5-chloro-4-phenyl-	other	OTH31	-	-	-	-	-	-	-	-	-	-	-	2.21±1.06
8-Azidoadenosine	other	OTH32	-	-	-	-	-	-	-	-	5±1.58	-	-	-
Aminoguanidine	other	OTH33	-	-	-	-	-	-	-	-	6.17±1.63	-	-	-
Crinamidine, 3-oxo-	other	OTH34	-	-	-	-	-	-	-	-	-	-	-	-
Cyclopentadienyltungstentricarbonyl bromide	other	OTH35	-	-	-	-	-	-	-	-	-	-	-	7.74±1.43
Cyclopropanecarbonitrile, 1,2-diphenyl-	other	OTH36	-	1.26±0.09	-	-	-	-	-	-	-	-	-	-
D-Glucose, 3,4,5,6-tetrakis-O-(trimethylsilyl)-, O-methyloxime, 2-[1-(trimethylsilyl)-1H-indole-3-acetate]	other	OTH37	-	-	-	-	1.64±0.07	-	-	-	-	-	-	-
D-Homo-24-nor-17-oxachola-20,22-diene-3,7,16-trione, 14,15:21,23-diepoxy-4,4,8-trimethyl-, (5.alpha.,13.alpha.,14.beta.,15.beta	other	OTH38	-	-	-	-	-	1.13±0.09	-	-	-	6.11±1.45	-	-
Diaziridine,3,3-dimethyl-	other	OTH39	-	-	-	-	3.76±1.23	-	-	-	-	-	-	-

diethyltriselenide	other	OTH40	-	-	3.55±1.02	-	-	-	-	-	-	-	-	-
Ethyl Chloride	other	OTH41	-	4.01±1.14	-	6.05±1.56	-	-	-	-	-	-	-	-
Hexa(trimethylsilyl)disilane	other	OTH42	-	-	-	-	-	-	-	-	3.24±1.04	-	-	-
Malonic acid, dihydrazide	other	OTH43	-	2.59±1.10	-	-	-	-	-	-	-	-	-	-
Methyl trans-2-octadecenoate	other	OTH44	-	-	-	-	-	-	-	2.53±1.78	-	-	-	-
Octadecane, 6-methyl-	other	OTH45	20.3±2.13	-	-	-	-	-	-	-	-	-	-	-
Piperidine, 4-benzyl-1-(3,5-dimethoxybenzoyl)-	other	OTH46	-	-	-	1.76±0.05	-	-	-	-	-	-	-	-
Pyridine, 2-(ethylthio)-3,6-diphenyl-	other	OTH47	-	-	-	-	-	-	-	-	-	2.82±0.47	-	-
Pyrimidine, tetrakis[(trimethylsilyl)oxy]-	other	OTH48	-	-	0.49±0.03	-	-	-	-	-	-	-	-	-
Quinazolin-4(3H)-one, 2-[2-(2-hydroxy-5-bromophenyl)ethenyl]-3-methyl-	other	OTH49	-	3.18±1.02	-	-	-	-	-	-	-	-	-	-
Succinic anhydride	other	OTH50	-	2.29±0.06	-	29.36±2.60	-	2.68±1.20	8.35±1.47	12.28±1.89	-	-	-	2.26±1.05
[1,1'-Binaphthalene]-2,2'-dithiol	sulfur	SUL1	-	-	-	-	-	-	-	-	13.4±1.45	-	-	-
[1,2,4]Triazole-3-thione, 5-isopropyl-2-phenyl-1,2-dihydro-	sulfur	SUL2	-	2.8±1.47	-	-	-	-	-	-	-	-	-	-
2-Methyl-1,3-oxathiolane	sulfur	SUL3	-	-	-	3.23±0.25	-	-	-	-	-	-	-	-
Butanoic acid, 4,4'-dithiobis[2-amino-, [S-(R*,R*)]-	sulfur	SUL4	1.7±0.23	-	-	-	-	-	-	-	-	-	-	-
Diethyl Phthalate	sulfur	SUL5	-	-	-	-	-	5.97±1.41	-	-	-	-	6.21±1.05	-
Hydrazinecarbothioamide, N-2-propenyl-	sulfur	SUL6	-	-	-	-	-	-	-	-	-	1.85±0.05	-	-
Hydrazinecarbothioamide, N-methyl-	sulfur	SUL7	2.66±0.48	-	-	-	-	5.96±0.28	1.02±0.01	6.6±0.36	-	-	-	-
hydrazinecarbothioamide, N-octadecyl-	sulfur	SUL8	-	-	-	-	-	-	-	-	1.6±0.03	1.75±0.01	-	-
hydrazinecarboxamide, 2-(aminothioxomethyl)-N-butyl-	sulfur	SUL9	-	-	8.97±1.45	-	-	-	-	-	-	-	-	-
Methanone, bis[4-(diethylamino)phenyl]-, O-methyloxime	sulfur	SUL10	-	-	-	-	-	-	-	-	-	-	3.49±1.03	-
Tetrahydro-1,3-oxazine-2-thione	sulfur	SUL11	-	-	-	-	-	-	-	-	0.44±0.04	-	-	-
**T=8°C**														
1,2-Benzenedicarboxylic acid, 3,4,5,6-tetrachloro-	acid	AC1	-	-	-	-	-	-	-	-	-	0.93±0.02	-	-
2-Azetidinecarboxylic acid	acid	AC2	-	4.49±1.03	-	-	-	-	-	-	-	-	-	-
2-Hexenedioic acid, 2,4-dichloro-5-oxo-	acid	AC3	-	-	-	-	-	-	-	-	-	2.75±1.06	-	-
4,5-Imidazoledicarboxylic acid	acid	AC4	-	-	8.34±1.04	-	-	-	-	-	-	-	-	-
N-[3,5-Dinitropyridin-2-yl]glutamic acid	acid	AC5	-	-	-	-	-	-	-	-	-	11.32±2.36	-	-
Naphthalene-2-carboxylic acid, 1,4-dihydro-1-oxo-4-diazo-	acid	AC6	-	-	-	-	-	-	-	-	-	-	6.24±	-
butanedioic acid, 2-cyano-2,3-dimethyl-	acid	AC7	9.98±1.56	0.97±0.05	-	9.08±1.04	1.87±0.03	8.05±1.45	3.98±1.03	4.49±1.47	-	0.31±0.01	1±0.01	-

Diphenylphosphinodithioic acid	acid	AC8	-	-	-	-	-	-	-	-	17.56±2.14	-	-	-
Glutaric acid, 3-chlorophenyl 2-	acid	AC9	1.48±0.25	-	-	-	-	-	-	-	-	-	-	-
hydrazinecarboxylic acid, 2-[(methylamino)thioxomethyl]-1-phenyl-	acid	AC10	-	-	-	-	1.27±0.14	-	-	-	-	-	-	-
propanoic acid, 3-[(4-hydroxy-1-phthalazinyl)oxy]-	acid	AC11	-	-	-	-	-	8.95±1.23	-	-	-	-	-	-
1,4-benzenediol, 2-(1-methylheptadecyl)-6-[(4-methylphenyl)thio]-	alcohol	AL1	3.51±1.05	-	-	-	-	-	-	-	-	-	-	-
1,4-Cyclohexanediol, 1,4-diphenyl-	alcohol	AL2	-	6.85±1.54	-	-	-	-	-	-	-	-	-	-
1,5-Naphthalenediol, 2,6-dibromo-	alcohol	AL3	-	-	-	-	-	-	-	-	-	2.66±1.05	-	-
2,2,2-Trichloro-1-(2-nitrophenylthioamino)ethanol	alcohol	AL4	-	-	-	0.36±0.03	-	-	-	-	-	-	-	-
1,4-Dioxane, 2,3-dichloro-	alkane	ALK1	2.55±1.04	-	-	-	-	-	-	-	-	-	-	-
Butane, 1,2,3,4-tetrabromo-	alkane	ALK2	-	-	-	-	-	-	-	-	-	-	1.85±0.06	-
Cyclohexanone, 2,6-bis[(4-azidophenyl)methylene]-	alkane	ALK3	-	-	-	-	-	7.77±1.48	-	-	-	-	-	-
Cyclohexanone, 6-methyl-3-(1-methylethyl)-2-(2-oxopropyl)-	alkane	ALK4	-	4.09±1.12	-	-	-	-	-	-	-	-	-	-
N,N-Dinitro-1,3,5,7-tetrazabicyclo[3,3,1]nonane	alkane	ALK5	-	-	-	4.01±1.53	-	-	-	-	-	-	-	-
p-Dioxane, methylene-	alkane	ALK6	-	-	-	-	-	-	-	-	-	3.82±0.56	-	-
1-Propene, 3-azido-	alkene	ALE1	-	-	8.65±1.36	-	-	-	-	-	-	-	-	-
Ethylene	alkene	ALE2	-	-	-	-	-	2.72±0.04	23.45±2.58	-	-	-	-	-
Naphthalene, 2-(4-cyanophenyl)-6-nonyl-	alkene	ALE3	-	-	-	-	4.22±1.05	-	-	-	-	-	-	-
4-Isothiazolecarboxamide, N-ethyl-3,5-bis(methylthio)-	amide	AM1	-	-	-	-	-	-	-	-	-	-	9.76±1.25	-
Acetamide, 2-[(1,3-dihydro-6-methyl-3-oxofuro[3,4-c]pyridin-4-yl)thio]-N-(4-ethylphenyl)-	amide	AM2	-	-	-	-	-	-	-	2.26±1.06	-	-	-	-
Acetamide, N-(2-hydroxy-1,1-dimethylethyl)-2-(1-methyl-3-phenylthio-2-indolyl)-	amide	AM3	-	-	2.31±0.25	-	-	-	-	-	-	-	-	-
Benzamide, 4-(5-bromo-2-thenylidenamino)-	amide	AM4	-	-	-	-	-	-	-	-	-	12.25±1.45	-	-
Dodecanamide, N-octadecyl-	amide	AM5	-	13.28±1.13	-	-	-	-	-	-	-	-	-	-
Formamide, N-(4,6-diamino-5-pyrimidinyl)-	amide	AM6	-	-	-	-	-	-	3.24±0.65	-	-	-	-	-
Hydrazinecarbothioamide, N-methyl-	amide	AM7	-	-	-	-	-	-	1.55±0.08	-	-	-	-	-
N-(4-Iodo-phenyl)-4-morpholin-4-ylmethyl-benzamide	amide	AM8	-	-	-	-	-	-	11.33±1.78	-	-	-	-	-
1,2-Ethanediamine, N,N'-bis(2,2,3,4,4-pentamethyl-1-phosphetanyl)-, P,P'-	amine	AMN1	-	-	-	-	-	-	-	-	1.44±0.058	-	-	-

dioxide														
1H-1,2,3-Triazol-5-amine, 4-phenyl-1-(3-pyridinyl)-	amine	AMN2	-	-	-	-	-	-	0.59±0.25	-	-	-	-	-
2-Naphthylamine, N-cyclohexyl-1,2,3,4-tetrahydro-	amine	AMN3	-	-	-	-	-	-	-	1.47±0.36	-	-	-	-
4H-1,2,4-Triazol-4-amine	amine	AMN4	-	3.35±1.06	-	-	-	-	-	-	-	-	-	-
Ethanamine, N,N-diethyl-2,2-bis[(trichloromethyl)thio]-	amine, sulfur	AMN5	12.15±2.36	-	-	-	-	-	-	-	-	-	-	-
1,3-benzenedisulfonyl dichloride, 4-methyl-	aromatic	AR1	-	-	-	-	-	-	-	-	-	2.97±0.14	-	-
4-Butyl-4'-methoxy-NNO-azoxybenzene	aromatic	AR2	-	-	-	-	-	-	-	-	12.37±1.56	-	-	-
5-Methoxy-2-[5-(4-methoxyphenyl)pyrimidin-4-yl]phenol	aromatic	AR3	-	-	12.71±1.85	-	-	-	-	-	-	-	-	-
Phenol, 4-(aminomethyl)-2-methoxy-	aromatic	AR4	-	-	-	-	1.88±0.09	-	-	-	19±	6.13±	-	-
Phenol, 4-[2-[2-(chloromethyl)-1,3-dioxolan-2-yl]ethyl]-, acetate	aromatic	AR5	-	-	-	-	-	-	1.72±0.08	-	-	-	-	-
Benzoic acid, 2-(phenylamino)-	aromatic acid	AR6	-	-	-	2.41±0.36	-	-	-	-	-	-	-	-
benzenamine, 4-(1,1-dioxido-4-thiomorpholinyl)-	aromatic amine	AR7	-	-	-	-	-	-	-	1.88±0.01	-	6.61±1.06	-	-
benzenamine, 4,4',4''-methylidynetris[2-methyl-	aromatic amine	AR8	-	-	-	-	-	3.61±0.58	-	-	-	-	-	-
Benzenamine, 4,4',4''-methylidynetris[N,N-dimethyl-	aromatic amine	AR9	10.4±1.67	7.44±1.56	-	-	-	-	-	-	-	-	-	-
Benzoic acid, 3-(2,3-dimethoxy-4,5-methylenedioxyphenyl)propyl ester	aromatic ester	AR10	-	-	-	-	-	-	-	-	4.11±0.25	-	-	-
Benzoic acid, 4-nitroso-, ethyl ester	aromatic ester	AR11	-	-	-	-	-	-	-	-	-	3.68±0.36	-	-
Acetic acid, hydrazide	azide	AZ1	-	-	-	-	-	-	-	-	-	7.65±1.47	-	-
N-(N-Methylformamidyl)-semithiocarbazide	azide	AZ2	-	-	-	-	2.35±0.05	-	-	-	-	-	-	-
Semioxamazide	azide	AZ3	-	-	1.12±0.01	-	-	-	-	-	-	-	-	-
[2-(Carbobenzyloxy)decahydroisoquinolin-3-yl]acetic acid, t-butyl ester	ester	EST1	-	-	-	-	5.23±1.58	-	-	-	-	-	-	-
1,4-Dihydro-1,4-ethenoanthracene-2,3-dicarboxylic acid, 9-phenyl-, dimethyl ester	ester	EST2	-	-	-	-	-	-	-	-	19.81±2.56	-	-	-
2,1-benzisoxazole-4,6,7-tricarboxylic acid, 1,3-dihydro-1-methyl-5-nitro-3-oxo-, trimethyl ester	ester	EST3	-	-	-	-	-	-	-	-	-	1.86±0.05	-	-
2,6-Pyridinedicarboxylic acid, hexyl octadecyl ester	ester	EST4	-	-	-	-	7.8±1.07	-	-	-	-	-	-	-
2-Propenoic acid, ethenyl ester	ester	EST5	-	5.51±1.13	-	-	-	-	-	-	-	-	-	-
3-(2-Amino-acetylamino)-3-phenyl-propionic acid, methyl ester	ester	EST6	-	-	-	-	-	-	-	-	-	-	7.63±1.52	-

5-Oxazolecarboxylic acid, 4-methyl-, ethyl ester	ester	EST7	8.83±1.58	-	-	-	-	-	-	-	-	-	-	-
Acetic acid, 2-(3',4'-dihydro-1'H-spiro[[1,3]dioxolane-2,2'-naphthalen]-1'-yl)ethyl ester	ester	EST8	-	-	-	-	-	-	-	-	-	-	5.63±1.05	-
Acetic acid, phenylhydrazonochloro-, ethyl ester	ester	EST9	-	-	-	-	-	1.44±0.06	-	-	-	-	-	-
Desoxy-isosteviol methyl ester	ester	EST10	-	10.41±2.03	-	-	-	-	-	-	-	-	-	-
Ethyl 3-methylsulfonyl-6-trifluoromethylphenanthrene-9-carboxylate	ester	EST11	-	-	-	-	-	-	1.83±0.41	-	-	-	-	-
Glycine, N,N-bis(2-ethoxy-2-oxoethyl)-, ethyl ester	ester	EST12	0.98±0.01	-	-	-	-	1.44±0.04	-	5.42±1.03	-	-	-	-
i-Propyl 5,9-hexacosadienoate	ester	EST13	-	-	-	-	-	-	-	-	-	-	2.91±0.01	-
l-Methionine, n-propargyloxycarbonyl-, nonyl ester	ester	EST14	-	-	-	-	-	-	-	-	-	2±0.14	-	-
Methyl (4-iodophenyl)pentadecanoate	ester	EST15	-	-	-	5.53±1.13	-	-	-	-	-	-	-	-
Methyl 5,8-diethoxyquinoxaline-2,3-dicarboxylate	ester	EST16	-	-	-	-	-	1.8±0.03	-	-	-	-	-	-
Propylene Carbonate	ester	EST17	-	-	-	-	-	-	-	-	-	0.41±0.01	-	-
Triethyl 1-cyano-3,3,4-pentanetricarboxylate	ester	EST18	-	15.91±1.87	-	-	-	-	-	-	-	-	-	-
(3,6-Dinitro-4-phenyl-quinolin-2-yl)-hydrazine	hydrazine	HYD1	1.58±0.06	-	-	-	-	-	-	-	-	-	-	-
Succinimide	imide	IMD1	-	-	-	-	-	-	2.18±0.02	-	-	-	-	-
1-(5-Hydroxy-3-pyridin-4-yl-5-trifluoromethyl-4,5-dihydropyrazol-1-yl)-2-methylpropan-1-one	ketone	KET1	-	-	-	-	-	-	1.66±0.01	-	-	-	-	-
1,4-Cyclohexanedione	ketone	KET2	-	5.19±1.09	-	-	-	-	-	-	-	-	-	-
1,6-Dioxaspiro[4.4]nonane-2,7-dione	ketone	KET3	-	-	-	-	-	-	-	-	-	-	11.43±1.95	-
2,4(1H,3H)-Pyrimidinedione, dihydro-3-methyl-	ketone	KET4	-	-	-	-	4.93±1.06	-	-	-	-	-	-	-
2,5-Pyrrolidinedione, 1-(2-methylene-3-butenyl)-	ketone	KET5	-	-	-	-	-	-	-	-	-	-	3.36±0.06	-
2,6-Bis(4-azidobenzylidene)-4-methylcyclohexanone	ketone	KET6	-	2.48±0.62	5.65±1.58	-	-	12.32±2.06	-	5.16±1.14	-	1.74±0.09	8.36±1.42	-
2-Nonacosanone	ketone	KET7	-	-	-	-	-	-	-	-	2.24±1.12	-	-	-
3H-phenothiazin-3-one, 7-(diethylamino)-	ketone	KET8	-	-	-	-	3.43±1.54	-	-	-	-	-	-	-
3-Penten-2-one, 4-[(1,6-dimethyl-2-phenyl-1H-indol-5-yl)amino]-	ketone	KET9	-	-	-	-	-	-	-	-	-	-	-	2.42±0.36
4H-Thiopyran-4-one, tetrahydro-	ketone	KET10	-	-	-	-	-	-	-	-	-	-	2.46±1.23	-
6-Oxabicyclo[3.1.0]hex-2-en-4-one, 1,2,3,5-tetraphenyl-	ketone	KET11	-	-	-	-	6.05±1.41	-	-	-	-	-	-	-

7,9-Dinitro-1,2,3,4-tetrahydro-benzo[e][1,4]diazepin-5-one	ketone	KET12	-	-	-	-	-	-	7.43±1.86	-	-	-	-	-
7-Methoxy-4-(7-methoxy-2H-1,3-benzodioxol-5-yl)-3,4-dihydro-1-benzopyran-2-one	ketone	KET13	-	-	-	2.62±0.03	-	-	-	-	-	-	-	-
9-Heptadecanone	ketone	KET14	-	-	-	-	-	-	-	-	-	-	2.55±1.36	-
Androst-1-ene-3,17-dione	ketone	KET15	-	-	-	7.8±1.12	-	-	-	-	-	-	-	-
Bis-(3-azido-4-methoxy-phenyl)methanone	ketone	KET16	-	-	6.67±1.23	-	-	-	-	-	-	-	-	-
Hexa-1,4-dien-3-one, 5-amino-6,6,6-trichloro-1-(2-thienyl)-	ketone	KET17	7.45±1.47	-	-	-	-	-	-	-	-	-	-	-
1,2,4-Triazine	nitrogen	N1	-	-	-	-	-	-	-	-	-	1.29±0.03	-	-
Acetonitrile, hydroxy-	nitrogen	N2	-	-	-	-	-	-	0.91±0.01	-	-	-	-	-
Cyacetacide	nitrogen	N3	-	-	-	-	0.44±0.01	-	-	-	-	-	-	-
Hydrazine	nitrogen	N4	-	-	-	-	-	-	19.38±2.14	-	-	-	-	-
Hydrazine, (2-methyl-1-propenyl)-	nitrogen	N5	-	-	-	-	-	-	-	-	9.43±1.56	-	-	-
1-(3,4-Diethoxybenzoyl)-6,7-diisopropxy-isoquinoline	other	OTH1	-	2.28±1.05	-	-	-	-	-	-	-	-	-	-
1,1'-Binaphthyl, 2,2'-diacetamido-	other	OTH2	-	4.77±1.14	-	-	-	-	-	-	-	-	-	-
1,1'-Biphenyl, 2,2',3,4,5'-pentachloro-	other	OTH3	-	-	-	5.91±1.23	-	-	-	-	-	-	-	-
1,5:2,4-Dimethanopentalen-3(2H)-one, 1,5,6,6,6a,7,8-heptachlorohexahydro-, (1.alpha.,2.beta.,3a.alpha.,4.beta.,5.alpha.,6a.alph	other	OTH4	-	-	-	-	-	-	-	-	-	-	-	9.44±1.36
1H,5H-benzo[ij]quinolizine, 2,3,6,7-tetrahydro-9-nitroso-	other	OTH5	-	-	-	-	5.93±1.25	-	-	-	-	-	-	-
1H-Pyrazole, 1-(9-borabicyclo[3.3.1]non-9-yl)-3,5-bis(1,1-dimethylethyl)-	other	OTH6	-	-	-	-	3.34±0.36	-	-	-	-	-	-	-
2(1H)-Phenanthrenone, 3,4,4a,9,10,10a-hexahydro-1,1,4a-trimethyl-, (4aS-trans)-	other	OTH7	-	-	-	-	-	7.48±1.29	-	-	-	-	-	-
2-(3-Methylthiophen-2-yl)-[1,2,4]triazolo[1,5-c]quinazoline	other	OTH8	-	-	-	-	-	-	-	-	-	-	15.4±1.84	-
2-(5-Ethyl-2-quinuclidinyl)-3-(2-hydroxyethyl)-5-methoxyindole	other	OTH9	-	-	-	-	-	1.59±0.06	-	-	-	-	-	-
2,4,6(1H,3H,5H)-Pyrimidinetrione, 5-[(3,4-dihydroxyphenyl)methylene]-1-(2-propenyl)-	other	OTH10	-	-	-	-	-	-	-	1.77±0.04	-	-	-	-
2,4-Bis(5-chlorothien-2-yl)-6-dicyanomethylene-2-methyl-1,2,3,6-tetrahydopyridin-5-carbonitrile	other	OTH11	0.92±0.01	-	-	-	-	-	-	-	-	-	-	-
2-Butene ozonide	other	OTH12	-	-	-	-	0.91±0.02	-	-	-	-	-	5.08±1.04	-
2-Butenoic acid, 2-methyl-,	other	OTH13	-	-	-	-	-	-	-	-	2.31±0.58	-	-	-

1,1a,1b,4,4a,5,7a,7b,8,9-decahydro-4a,7b-dihydroxy-1,1,6,8-tetramethyl-3-[[(2-methyl-1-oxo-2-buteny														
2-cyclopenten-1-one, 4-hydroxy-5-(1-naphthalenylmethylene)-3,4-diphenyl-	other	OTH14	-	-	1.47±0.87	-	-	-	-	-	-	-	-	-
2-Ethoxy-5-(1,2,4-triazol-4-yl)aniline	other	OTH15	-	-	-	-	-	-	-	-	-	5.6±1.41	-	-
2H-1,4-Benzodiazepin-2-one, 3-(acetyloxy)-7-bromo-5-(2-chlorophenyl)-1,3-dihydro-	other	OTH16	-	-	-	-	-	-	-	-	-	-	-	8.43±1.56
3-(2-Oxo-1,3,4-oxathiazol-5-yl)propanenitrile	other	OTH17	-	5.1±1.58	-	-	-	-	-	-	-	-	-	-
3-Ethoxy-1,1,1,5,5,5-hexamethyl-3-(trimethylsiloxy)trisiloxane	other	OTH18	-	14.12±1.87	-	-	-	-	-	-	-	-	-	-
4,4-Dimethyl-2-oxo-6-pentylsulfanyl-1,2,3,4-tetrahydro-pyridine-3,5-dicarbonitrile	other	OTH19	-	-	11.99±1.58	-	-	-	-	-	-	-	-	-
4,4'-Methylenebis(thiophene-3-methanol), 2,2',5,5'-tetramethyl-, diethyl ether	other	OTH20	-	-	-	-	-	-	0.81±0.01	-	-	-	-	-
4-[3-(3,4-Dimethoxyphenyl)-[1,2,4]triazolo[3,4-b][1,3,4]thiadiazol-6-yl]-1,2,3-thiadiazole	other	OTH21	-	-	-	-	-	5.78±1.41	-	-	-	-	-	-
4-Methoxymethyl-5-phenyl-2-(1-phenylthio-ethyl)-4,5-dihydro-oxazole	other	OTH22	-	-	-	-	0.43±0.02	-	-	-	-	-	-	-
5,8-Methano-1H-[1,2,4]triazolo[1,2-a]pyridazine-1,3(2H)-dione, 5,6,7,8-tetrahydro-2,6-diphenyl-	other	OTH23	-	-	-	-	2.2±1.04	-	-	-	-	-	-	-
8-Azahypoxanthine	other	OTH24	-	-	-	-	-	8.43±1.85	-	-	-	-	6.46±1.06	-
9,10-Epoxy-1,2,3,9,9a,10-Hexahydro-9,9a,10-triphenylanthracene	other	OTH25	-	-	-	-	-	3.96±0.08	-	-	-	-	-	-
Aconitane-1,7,8,14-tetrol, 20-ethyl-6,16-dimethoxy-4-(methoxymethyl)-, 14-acetate, (1.alpha.,6.beta.,14.alpha.,16.beta.)-	other	OTH26	14.73±1.74	-	-	-	-	-	-	-	-	-	-	-
Adenosine, N-(4-hydroxy-3-methylbutyl)-	other	OTH27	-	-	-	-	8.4±1.41	-	-	-	-	-	6.84±1.05	-
CH3CHClCN	other	OTH28	-	3.73±1.74	-	-	-	-	-	-	-	-	-	-
Cholestan-26-oic acid, 3,7-dihydroxy-, methyl ester, (3.alpha.,5.beta.,7.alpha.)-	other	OTH29	15.57±1.85	-	-	-	-	-	-	-	-	-	-	-
Cyano(ethoxycarbonyl)methoxyiminomethane	other	OTH30	1.4±0.08	-	-	-	-	-	-	-	-	-	-	-
Cyclopentaneheptanoic acid, 3-hydroxy-2-(3-hydroxy-1-octenyl)-5-oxo-, methyl ester, stereoisomer	other	OTH31	-	-	2.42±0.10	-	-	-	-	-	-	-	-	-
D-Homo-24-nor-17-oxachola-20,22-diene-3,7,16-trione, 14,15:21,23-diepoxy-4,4,8-trimethyl-, (5.alpha.,13.alpha.,14.beta.,15.beta	other	OTH32	-	-	-	1.73±0.21	-	-	1.46±0.13	-	-	-	-	-

dimethyl pentacyclo[6.6.6.0(2,7).0(9,14).0(15,20)]icosane-1,8-dicarboxylate	other	OTH33	-	-	-	-	-	2.54±1.23	-	-	-	-	-	-
Dimethyl phosphite	other	OTH34	-	-	-	-	-	6.83±1.57	-	-	-	-	-	-
Equilin, O-ethyloxime	other	OTH35	-	-	-	16.76±1.29	-	-	-	-	-	-	-	-
Ethyl Chloride	other	OTH36	-	-	-	-	-	7.49±1.25	-	-	-	-	-	-
Hexaphenyldisiloxane	other	OTH37	-	-	-	-	5.92±1.45	-	-	-	-	-	-	-
Hydrazine, N-[1-(pyrid-2-yl)ethylidene]-N'-[(3-azabicyclo[3.2.2]nonan-3-yl)-(bromocopperothio)methylidene]-	other	OTH38	-	-	-	-	-	0.67±0.06	-	-	-	-	-	-
n-Butyryl-l-methionine anilide	other	OTH39	2.16±0.56	-	-	-	-	-	-	-	-	-	-	-
p,p'-Benzylidenebis(N,N-dimethylaniline)	other	OTH40	-	-	-	-	-	-	-	5.55±1.47	-	-	-	-
Pennogenin, 24-hydroxy-	other	OTH41	-	3.26±1.14	-	-	-	-	-	-	-	-	-	-
Pentaborane(11)	other	OTH42	-	-	13.9±1.58	-	-	-	-	-	-	-	-	-
Pentagermane	other	OTH43	-	-	-	-	-	-	-	18.34±1.96	-	-	-	-
Pyridine, 3-methyl-2-(2-nitrophenyl)-	other	OTH44	-	-	-	-	0.64±0.03	-	-	-	-	-	-	-
pyrimidine, 2-hydrazinyl-4,6-dimethoxy-	other	OTH45	-	-	6.93±1.05	-	-	1.53±0.06	4.59±1.54	10.72±1.86	-	-	-	-
quinoline, 2-[3-(2,4-dinitrophenyl)-2-propen-1-ylidene]-1-ethyl-1,2-dihydro-	other	OTH46	-	-	-	-	-	-	-	-	-	15.98±1.36	-	-
Spiro(isoquinoline-1,2'-indene),1,2,3,4,2',3'-hexahydro-6'-acetoxy-7'-ethoxy-6,7-dimethoxy-2-methyl-1'-oxo-	other	OTH47	-	-	-	3.81±0.05	-	-	-	-	-	-	-	-
Spiro[acridine-9(10H),1'-cyclohexane]	other	OTH48	-	-	-	-	-	-	-	37.7±2.41	-	-	-	-
Spiro[isoquinoline-1,2'-indene],1,2,3,4,2',3'-tetrahydro-7,6'-diacetoxy-7'-ethoxy-6-methoxy-2-methyl-1'-oxo-	other	OTH49	-	-	-	-	-	-	-	-	-	-	-	18.38±2.13
Stannane, dibutylmethyl(1-methylethyl)-	other	OTH50	-	-	-	15.54±1.12	-	-	-	-	-	-	-	-
Stannane, diiododiphenyl-	other	OTH51	-	-	-	-	-	-	-	-	3.41±1.02	-	-	-
Succinic anhydride	other	OTH52	-	-	-	-	1.51±0.21	1.59±0.36	-	-	-	-	-	-
1,2-Oxathiolane, 2,2-dioxide	sulfur	SUL1	-	-	-	-	-	-	-	-	-	-	-	30±2.36
1,3,5,7-Tetrathiocane	sulfur	SUL2	-	0.89±0.01	-	-	-	-	-	-	-	-	-	-
benzothiazole, 2-(methylsulfinyl)-	sulfur	SUL3	-	-	-	-	-	-	-	-	-	-	0.69±0.01	-
Perhydroindole, 3-methyl-1-[(4-methylphenyl)sulfonyl]	sulfur	SUL4	-	-	-	13.88±1.48	-	-	-	-	-	-	-	-
Thiophene, tetrahydro-, 1,1-dioxide	sulfur	SUL5	-	-	-	-	-	-	-	-	-	0.57±0.03	-	-
1-(3,4-Diethoxybenzyl)-6,7-diisopropoxy-isoquinoline	sulfur	SUL6	-	-	-	4.41±1.13	-	-	-	-	-	-	-	-

1-Propene, 3-azido-	sulfur	SUL7	-	-	-	-	-	3.41±1.05	-	-	-	-	-	-
**T=4°C**														
2-((2 Butoxyethoxy)carbonyl) benzoic acid	acid	AC1	-	-	1.31±0.12	-	-	-	-	-	-	-	-	-
3-(2-Aminoethyl)-7-methoxy-5-nitro-1H-indole-2-carboxylic acid	acid	AC2	4.1±1.02	-	-	-	-	-	-	-	-	-	-	-
3,4-Methylenedioxyphenyllactic acid, O,O'-bis(trimethylsilyl)-	acid	AC3	-	-	-	-	-	-	-	-	12.1±1.46	-	-	-
4-[2-Hydroxyethyl)thio]benzoic acid	acid	AC4	-	-	-	-	-	5.68±1.16	-	-	-	-	-	-
Barbituric acid, 5-allyl-5-phenyl-	acid	AC5	-	-	-	-	-	-	-	-	-	3.27±1.58	-	-
Glycyl-dl-aspartic acid	acid	AC6	-	-	-	-	-	-	13.92±1.87	-	-	-	-	-
Malonic acid, 2-methyl-2-pentyl-	acid	AC7	-	-	-	-	-	-	-	-	-	-	-	3.99±1.36
Propanoic acid, 2-chloro-	acid	AC8	-	-	-	-	-	2.47±0.05	-	-	-	-	-	
1,2-Naphthalenediol, 1,2,3,4-tetrahydro-1-phenyl-, cis-	alcohol	AL1	-	-	-	-	-	-	-	-	-	4.68±1.05	-	-
1,4-benzenediol, 2,5-bis [(methylamino) methyl]-3,6 dioctyl-	alcohol	AL2	-	-	-	-	-	-	0.83±0.01	-	-	-	-	-
4,8,14-Trimethyl-11-isopropyltricyclo[7.5.0.0(10,14)]tetradec-4-en-8-ol	alcohol	AL3	-	-	-	-	-	-	-	-	7.81±1.06	-	-	-
6,11-Dihydrodibenzo(b,E)thiepin-11-ol	alcohol	AL4	14.69±1.84	-	-	-	-	-	-	-	-	-	-	-
2-(3,4,5-Trimethoxyphenyl)pyrimidine-5-carbaldehyde	aldehyde	ALD1		-	-	-	-	-	-	-	-	-	1.27±	
3-(Ehtylthio)propanal	aldehyde	ALD2	-	-	-	3.35±1.16	-	-	-	-	-	-	-	-
Cyclobutane	alkane	ALK1	-	-	-	-	-	-	-	-	-	-	-	14.03±1.42
Distannoxane, hexabutyl-	alkane	ALK2	-	-	-	-	-	-	-	-	-	-	-	0.96±0.05
Ethane	alkane	ALK3	-	-	-	-	-	-	-	-	-	-	1.05±0.04	
Ethane, diazo-	alkane	ALK4	-	-	-	-	-	-	-	-	0.84±0.01	-	-	-
Pentane, 1,1'-[methylenebis(oxy)]bis[2,2,3,3,4,4,5,5-octafluoro]-	alkane	ALK5	6.8±1.87	-	-	-	-	-	-	-	-	-	-	-
1,1'-Diacetoxy-4,4'-dichloro-5,5',8,8'-tetramethoxy-6,6'-dimethyl-2,2'-binaphthalene	alkene	ALE1	-	-	-	-	-	-	-	1.43±0.36	-	-	-	-
1,3,4-Hexatriene, 3-methoxy-	alkene	ALE2	-	-	-	-	-	-	3.48±1.04			-	-	-
1,3-Cyclopentadiene, 1,2,3,4-tetrachloro-5,5-dimethoxy-	alkene	ALE3	-	-	-	-	-	-	-	-	-	-	-	17.65±1.87
13-Methoxymethoxy-14-(2-methoxyphenyl)tricyclo[8.2.2.2(4.7)]hexadeca-1(13),4(16),5,7(15),10(14),11-hexaene	alkene	ALE4	-	-	-	-	-	-	-	-	-	5.32±1.25	-	-
1-Propene, 3-azido-	alkene	ALE5	-	9.07±1.45	-	-	-	-	-	-	-	-	-	-

2-Butene	alkene	ALE6	-	-	6.26±1.65	-	-	-	-	-	-	-	-	-
2-Butene ozonide	alkene	ALE7	-	-	-	-	-	-	-	-	-	-	-	4.33±1.24
Ethylene	alkene	ALE8	-	26.23±2.04	-	-	-	-	-	-	-	-	-	-
Pentacene, 5,6,7:12,13,14-hexathio-	alkene	ALE9	-	-	-	13.02±1.14	-	-	-	-	-	-	-	-
Oxamide, N-cyclopentyl-N'-(3-methoxypropyl)-	amide	AM1	-	-	-	-	-	-	-	1.09±0.05	-	-	-	-
Propanamide, 3-cyclopentyl-N-pentyl-	amide	AM2	-	-	-	-	-	-	4.82±0.23		-	-	-	-
1,2-ethanediamine, N1,N2-bis(4,5-dichloro-2-nitrophenyl)-	amine	AMN1	-	-	-	1.16±0.04	-	-	-	-	-	-	-	-
1,3,4-Oxadiazol-2-amine, 5-phenyl-	amine	AMN2	-	-	-	-	-	5.08±1.14	-	-	-	-	-	-
1-Butanamine, N-(1-methylethyl)-	amine	AMN3	13.54±1.56		-	-	-	-	-	-	-	-	-	-
2,5-Dichlorobenzylamine	amine	AMN4	-	6.84±1.06	-	-	-	-	-	-	-	-	-	-
2-Naphthylamine, N-cyclohexyl-1,2,3,4-tetrahydro-	amine	AMN5	-	-	-	-	-	-	-	-	8.85±1.15	-	-	12.18±1.46
Ethenamine, N-methylene-	amine	AMN6	-	-	-	9.83±1.48	-	-	-	-	-	-	-	-
Propylamine, N-acetyl-3-[2-acetyl-4,5-dimethoxyphenyl]-	amine	AMN7	-	-	-	-	-	-	-	-	3.45±0.36	-	-	-
5-Methoxy-2-(naphthalen-2-ylazo)-phenol	aromatic	AR1	-	-	1.45±1.02	-	-	-	-	-	-	-	-	-
Benzene, (azidomethyl)-	aromatic	AR2	-	-	-	-	-	-	2.64±0.03	-	-	-	-	-
Benzene, 1-(dimethoxymethyl)-2-amino-	aromatic	AR3	-	-	-	-	-	-	-	-	1.49±0.04	-	-	-
Benzene, 1,2,4,5-tetrachloro-3-nitro-	aromatic	AR4	-	-	-	-	-	-	-	0.8±0.01	-	-	-	-
benzenamine, 4-(1,1-dioxido-4-thiomorpholinyl)-	aromatic	AR5	-	-	-	-	-	3.53±1.06	-	-	-	-	-	-
Benzenamine, 4,4',4"-methylidynetris[N,N-dimethyl-	aromatic	AR6	-	-	-	-	-	-	-	1.92±0.45	-	-	-	-
benzenamine, 4-[bis(2,4,6-trimethylphenyl)boryl]-N,N,3,5-tetramethyl-	aromatic	AR7	-	-	-	-	-	-	-	0.66±0.10	-	-	-	-
Benzeneethanamine, 3-isothiocyanato-N-[2.alpha.-[1-tetrahydropyrrolyl]-	aromatic	AR8	-	-	-	-	-	-	-	-	-	-	2.14±0.56	-
Benzoic acid, 2-(ethylthio)-, ethyl ester	aromatic	AR9	-	4.12±1.05	-	-	-	-	-	-	-	-	-	-
Benzoic acid, 2,5-dichloro-3,6-dimethoxy-, methyl ester	aromatic	AR10	-	-	9.21±1.75	-	-	30.22±2.34	-	-	3.08±1.20	-	-	-
Benzoic acid, 4-(1-azepinyl)azo-, ethyl ester	aromatic	AR11	1.34±0.25	-	-	-	-	-	-	-	-	-	-	-
Benzo[e][1]-benzothiophene, 4-(2-pyridylcarbonyl)-	aromatic	AR12	4.67±1.06	-	-	-	-	-	-	-	-	-	-	-
butanedioic acid, 2-cyano-2,3-dimethyl-, diethyl ester	aster	ASR1	-	-	-	-	-	-	-	23.53±1.58	-	-	-	-
Acetic acid, hydrazide	azide	AZ1	-	-	2.03±0.04	-	-	-	-	1.95±0.01	-	-	-	-

[1,1'-Bicyclohexyl]-4-carboxylic acid, 4'-pentyl-, 4-pentylphenyl ester	ester	EST1	-	-	1.65±0.65	-	-	-	-		-	-	-	-
1H-Indole-2-carboxylic acid, 3-acetylamino-5-fluoro-1-(2-oxopropyl)-, methyl ester	ester	EST2	-	-	-	-	-	13.04±1.87	-	-	-	-	-	5.08±0.25
2-(2-Hydroxy-3-phenyl-butyrylamino)-3-phenyl-propionic acid, methyl ester	ester	EST3	-	-	-	-	-	-	-	-	-	-	52.62±2.58	-
2,6-Pyridinedicarboxylic acid, di(3-methylbutyl) ester	ester	EST4	-	-	-	-	2.52±0.29	-	-	-	-	-	-	-
2-Indolizinecarboxylic acid, methyl ester	ester	EST5	-	-	-	-	-	-	1.95±0.02	-	-	-	-	-
3-Phenylpropionic acid, 2-methyloct-5-yn-4-yl ester	ester	EST6	-	-	-	5.73±0.47	-	-	-	-	-	-	-	-
4-Nitrobenzylidenenemalonic acid, diethyl ester	ester	EST7	-	6.56±1.14	-	-	-	-	-	-	-	-	-	-
5-Octadecenoic acid, methyl ester	ester	EST8	-	-	-	-	-	-	13.05±1.85	-	-	-	-	-
diethyl 2-methylphenylphosphonate	ester	EST9	-	-	-	-	-	-	-	-	-	-	0.36±0.01	-
Diethylmalonic acid, bis(trimethylsilyl)-ester	ester	EST10	-	-	-	-	-	-	-	-	5.93±1.09	-	-	-
Ethyl 2-cyano-3,7,11,15-tetramethyl-2-hexadecenoate	ester	EST11	-	-	4.16±1.05	-	-	-	-	-	-	-	-	-
Glycine, N,N-bis(2-ethoxy-2-oxoethyl)-, ethyl ester	ester	EST12	-	-	-	-	3.08±0.06	-	-	-	-	-	-	-
Heneicosanoic acid, isopropyl ester	ester	EST13	-	-	-	-	-	3.07±0.47	-	-	-	-	-	-
Hydrazinecarboxylic acid, ethyl ester	ester	EST14	1.88±0.41	-	-	-	-	-	-	-	-	-	-	-
l-Phenylalanine, N-(2-methoxyethoxycarbonyl)-, pentyl ester	ester	EST15	-	-	-	-	-	-	-	-	-	5.61±0.69	-	-
Succinic acid, butyl 2-methyl-3-nitrobenzyl ester	ester	EST16	-	-	-	-	-	2.41±0.23	-	-	-	-	-	-
Succinic acid, diamide, N,N,N',N'-tetraheptyl-	ester	EST17	-	-	-	2.27±0.03	-	-	-	-	-	-	-	-
Sulfurous acid, hexyl octyl ester	ester	EST18	-	-	-	-	-	-	3.24±0.42	-	-	-	-	-
Terephthalic acid, heptyl 3-methyl-5-methoxypentyl ester	ester	EST19	-	-	-	-	-	-	-	1.65±0.02	-	-	-	-
Terephthalic acid, monoamide, N-(2-ethylphenyl)-, butyl ester	ester	EST20	7.22±1.96	-	-	-	-	-	-	-	-	-	-	-
1,2-Oxathiolane, 2,2-dioxide	ether	ETH1	-	-	-	-	-	-	-	-	-	-	-	2.68±1.06
2,2',4,4'-Tetranitrodiphenylsulfoxide	ether	ETH2	-	-	-	-	-	-	-	-	-	-	-	3.45±1.05
2-Amino-3-benzyl-5-(4-methoxyphenyl)pyrazine-1-oxide	ether	ETH3	-	-	-	13.18±1.85	-	-	-	-	-	-	-	
Decachlorodiphenyl ether	ether	ETH4	-	-	-	15.35±2.45	-	-	-	-	-	-	-	-
Trimethylene oxide	ether	ETH5	-	2.85±0.65	-	-	-	-	-	-	-	-	-	-
1,2-Didehydrocrinan-3-one	ketone	KET1	-	-	-	-	0.92±0.05	-	-	-	-	-	-	-
1,4-anthracenedione, 5,6,7,8-tetrachloro-	ketone	KET2	-	-	-	-	-	-	-	-	6.53±0.68	-	-	-

2,4(1H,3H)-Pyrimidinedione, 1,3,6-trimethyl-	ketone	KET3	-	-	-	-	-	-	-	-	-	1.5±0.06	-	-
2,4(1H,3H)-Pyrimidinedione, dihydro-3-methyl-	ketone	KET4	8.15±1.01	-	-	-	-	-	-	-	-	-	-	-
2,5-Pyrrolidinedione, 1-hydroxy-	ketone	KET5	-	-	-	-	-	-	-	-	-	-	22.79±1.85	-
2,5-Pyrrolidinedione, 1-methyl-	ketone	KET6	-	-	-	-	-	2.4±0.25	-	0.64±0.01	-	-	-	-
2,6-Bis(4-azidobenzylidene)-4-methylcyclohexanone	ketone	KET7	-	-	-	-	-	-	-	-	-	-	-	1.03±0.03
2H-1-Benzopyran-2-one, 4-(1H-indol-4-yl)-5,6,7-trimethoxy-	ketone	KET8	-	-	0.47±0.01	-	-	-	-	-	-	-	-	-
3-(2-Hydroxyethyl)-2-oxazolidinone	ketone	KET9	-	-	2.22±0.09	-	-	1.41±0.02	-	-	-	-	-	-
4H-1-Benzopyran-4-one, 2-(3,4-dimethoxyphenyl)-3,7-dimethoxy-	ketone	KET10	-	18.23±	-	-	2.59±0.35	-	-	6.63±1.12	5.43±1.01	-	-	8.08±1.03
4-thiazolidinone, 3-ethyl-5-[2-(3H-[1,2,4]thiadiazolo[4,3-a]pyridin-3-ylidene)ethylidene]-2-thioxo-	ketone	KET11	-	-	-	-	-	-	2.02±0.03	-	-	-	-	-
9,10-Anthracenedione, 1,5-dichloro-	ketone	KET12	-	-	-	-	-	0.44±0.01		-	-	-	-	-
Bis-(3-azido-4-methoxy-phenyl)methanone	ketone	KET13	-	-	-	-	1.71±0.21			-	-	-	-	-
ethanone, 2-(7-chloro-4-phenyl-1H-1,5-benzodiazepin-2-yl)-1-phenyl-	ketone	KET14	9.49±1.46	-	-	-	-	-	-	-	-	-	-	-
(2-Selenyl-3-benzo[b]thienylidene)-4-methylaniline	nitrogen	N1	-	-	-	-	-	-	-	-	-	-	-	1.81±0.05
1,2,4,5-Tetrazine	nitrogen	N2	-	-	-	-	-	-	15.52±1.85	-	-	-	-	-
2H-Tetrazole, 2-methyl-	nitrogen	N3	-	-	-	-	2.63±0.14	-	-	-	-	-	-	-
Acetonitrile, hydroxy-	nitrogen	N4	-	-	-	-	-	-	-	-	-	-	1.17±0.06	-
Hydrazine, (1,1-dimethylethyl)-	nitrogen	N5	12.95±1.65	-	-	-	-	-	-	-	-	-	-	-
Hydrazine, (2-methyl-1-propenyl)-	nitrogen	N6	-	-	-	-	-	-	18.21±1.78	-	-	-	-	-
(5,6,7-Triacetoxy-4b,8-dimethyl-2-oxo-2,3,4,4a,4b,5,6,7,8,8a,9,10-dodecahydro-phenanthren-1-yl)-acetic acid, methyl ester	other	OTH1	-	-	-	-	-	-	-	-	-	-	2.04±0.69	-
1-(6-Coumarinyl)-3-methyl-3-(2-methylphenyl)guanidine	other	OTH2	-	-	-	-	-	-	-	-	-	-	-	2.36±0.03
1,1'-Biphenyl, 2,2',3,3',5,6,6'-heptachloro-	other	OTH3	-	-	-	-	-	-	-	-	-	21.34±2.56	-	-
1,1'-Biphenyl, 2,2',3,4,4',5'-hexachloro-	other	OTH4	-	-	6.81±1.12	-	49.81±2.56	1.5±0.01	-	-	6.06±1.06	-	1.32±0.03	1.22±0.50
1,1-Cyclopropanedicarbonitrile, 2-methyl-	other	OTH5	-	-	-	-	-	-	-	-	-	-	0.8±0.01	
1,2,3,6,7,8-Hexachlorodibenzo-p-dioxin	other	OTH6	-	-	-	-	19.69±1.45	1.97±0.23	-	7.86±1.24	-	-	-	-
1,3,5,7,9-Pentavinyl-1,3,5,7,9-pentabutoxycyclopentasiloxane	other	OTH7	-	-	-	-	-	-	-	0.28±0.06	-	-	-	-
1,3-Dithiane-4,6-dicarboxylic acid, 5-(4-methoxyphenyl)-, 1,1,3,3-tetraoxide,	other	OTH8	-	-	-	-	-	-	-	-	-	2.81±1.02	-	-

dimethyl ester														
1,4-Dioxaspiro[4.5]decane, 2-(trimethylsilyloxymethyl)-	other	OTH9	-	-	-	-	-	-	-	-	-	10.61±1.85	-	-
14-Acetoxy-arteether	other	OTH10	-	-	-	-	-	-	-	3.49±0.36	-		-	-
1-Diphenylethenylsilyloxydodec-9-yn	other	OTH11	-	-	-	-	-	-	6.79±0.65		-	-	-	-
1H-Pyrazole, 4-bromo-	other	OTH12	-	-	-	-	-	-	-	-	-	-		3.42±0.21
1-Methyl-1-phenyltetrachlorocyclotriphosphazene	other	OTH13	-	-	-	4.85±1.12	-	-	-	-	-	-	-	-
1-Naphthacenecarboxylic acid, 2-ethyl-1,2,3,4,6,11-hexahydro-2,4,5,7,10-pentahydroxy-6,11-dioxo-, methyl ester, [1R-(1.alpha.,2	other	OTH14	-	-	-	5.23±0.85	-	-	-	-	-	-	-	-
2,4,5-Trihydroxypyrimidine	other	OTH15	-	-	-	-	-	2.57±0.24	-	-	-	-	-	-
3-(2,2-Dimethyl-4,6-dioxo-1,3-dioxan-5-ylidene)amino-4-carbethoxy-pyrazole	other	OTH16	-	-	-	-	-	-	-	-	-	2.93±0.21	-	-
3,5,6,7,7,8-Hexachloro-5,6,7,8-tetrahydro-S-triazolo[4,3-a]pyridine	other	OTH17	-	-	7.37±1.41	-	-	-	-	-	-	-	-	-
3-Methyl-1,2-diazirine	other	OTH18	-	-		-	-	3.1±0.65	-	-	-	-	-	-
5-Azido-desoxythymidine	other	OTH19	-	-	5.54±0.64	-	-	-	-	-	-	-	-	-
6,10-Methano-19-norandrost-4-ene-3,17-dione, 6-methoxy-	other	OTH20	-	-	-	-	-	-	-	-	-	-	-	3.41±0.03
6,16-Etheno-13,9-nitrilo-9H-cyclotrideca[b]naphthalene, 7,8,14,15-tetrahydro-8,14-bis(methylthio)-	other	OTH21	-	-	-	-	-	-	-	6.38±0.54	-	-	-	-
6-Aminotetrazolo(b)pyridazine	other	OTH22	-	-	-	-	17.05±1.42	-	-			-	0.4±0.01	2.12±0.06
6-Hydrazinotetrazolo(b)pyridazine	other	OTH23	-	-	-	-	-	-	-	11.59±1.24	7.35±0.54	-		
7,7'-Dihydroxy-8,8'-dimethoxy-3,3'-dimethyl-2,2'-binaphthalene-1,1',4,4'-tetrone	other	OTH24	-	-	-	-	-	-	-	-	-	-	-	7.52±1.02
9-Borabicyclo[3.3.1]nonane, 9-[[ethyl[1-ethyl-2-(trimethylgermyl)-1-propenyl]boryl]oxy]-, (Z)-	other	OTH25	-	-	-	-	-	-	-	-	-	-	1.55±0.05	-
Aminoglyoxime, N,N'-bis(2-chlorophenyl)-	other	OTH26	-	-	-	-	-	-	2.76±0.12	-	-	-	-	-
Bicyclo[2.2.1]heptane, 3-methylene-2-(3-phenylprop-1-en-2-yl)-	other	OTH27	-	-	-	-	-	-	-	-	-	-	4.61±0.54	-
Bis(2-phosphinoethyl)amine, P,P'-bis[tetracarbonyl-iron(0)]	other	OTH28	-	-	-	-	-	1.93±0.02	-	-	-	-	-	-
Bis(tert-butyldimethylsilyl) 2,3-bis((tert-butyldimethylsilyl)thio)succinate	other	OTH29	-	-	-	-	-	-	-	-	2±0.04	-	-	-
Butyrolactone	other	OTH30	-	-	-	-	-	-	-	-	-	-	0.56±0.01	
CH3CHClCN	other	OTH31	-	-	-	-	-	-	-	-	-	-		2.14±0.21
Cyclohexane, 1R-acetamido-4-cis-acetoxy-2,3-cis-epoxy-	other	OTH32	-	-	-	-	-	-	-	-		8.67±1.15	-	-

Cyclohexanecarboxamide, N-[2'-(3-methoxy-1-propanoyl)phenyl]-	other	OTH33	-	7.5±1.12	-	-	-	-	-	-	-	-	-	-
Cyclopentasiloxane, decamethyl-	other	OTH34	4.68±0.54	-	-	-	-	-	-	-	-	-	-	-
Dicarbadodecaborane-c,c'-bis(propanenitrile)-	other	OTH35	-	-	-	-	-	-	-	-	-	-	0.29±0.08	-
Glycine, N-[N-[N-[N-[cis-4-hydroxy-1-[N-[N-[(3-hydroxy-2-pyridinyl)carbonyl]-L-threonyl]-D-leucyl]-D-prolyl]-N-methylglycyl]-N,	other	OTH36	-	-	-	2.12±0.21	-	-	-	-	-	-	-	-
L-Serine, N-[N-(1-oxohexyl)glycyl]-, methyl ester, hexanoate (ester)	other	OTH37	7.3±1.35	-	-	-	-	-	-	-	-	-	-	-
Malonic acid, (9,10-anthrylenedimethylene)bis[methyl-, tetraethyl ester	other	OTH38	-	-	-	2.87±0.36	-	-	-	-	-	-	-	-
Phenanthro[1,2-b]furan-10,11-dione, 6,7,8,9-tetrahydro-6-(hydroxymethyl)-1,6-dimethyl-, (-)-	other	OTH39	-	-	10.55±1.41	-	-	-	-	-	-	-	-	-
Propanedinitrile, propylidene-	other	OTH40	-	-	-	-	-	3.22±0.74	-	-	-	-	-	-
Pyrimidine 2-amino-5-chloro-4,6,-bis[dichloromethyl]-	other	OTH41	-	-	-	-	-	-	3.85±0.24	-	-	-	-	
Pyrimidine, 2-(4'-heptyl[1,1'-biphenyl]-4-yl)-5-hexyl-	other	OTH42	-	-	-	-	-	-	-	-	-	-	-	0.41±0.01
Pyrrolidine-2-methanol, N-(dicyclohexylphosphino)-O-(dicyclopentylphosphino)-	other	OTH43	-	-	0.98±0.4	-	-	-	-	-	-	-	-	-
Sebacic acid, 2,6-dimethylnon-1-en-3-yn-5-yl propyl ester	other	OTH44	-	-	4.66±0.56	-	-	-	-	-	-	-	-	-
Succinic anhydride	other	OTH45	-	-	-	-	-	-	-	-	-	-	1.2±0.14	-
t-Butyl-(3-methylbut-3-enyloxy)diphenylsilane	other	OTH46	-	-	-	-	-	-	-	1.23±0.54	-	-	-	-
Thiophene-2-carboxylic acid, 3-(2-tert-butylsulfonyl-2-cyanoethenylamino)-, methyl ester	other	OTH47	-	-	-	-	-	0.32±0.02	-		-	-	-	-
Thiophene-2-carboxylic acid, 5-methylsulfonyl-4-nitro-	other	OTH48	-	16±1.87	-	-	-	15.64±1.95	-	2.68±0.42	-	-	1.46±0.13	-
1-(O-Methoxyphenyl)-3-phenyl-2-thiourea	sulfur	SUL1	-	-	-	1.15±0.65	-	-	-	-	-	-	-	-
1,2-Benzisothiazole, 3-[(4-nitrophenyl)methoxy]-, 1,1-dioxide	sulfur	SUL2		-	-		-	-	-	-	-	-	-	2.13±0.54
1,3-Dithiolane, 2,2-diphenyl-	sulfur	SUL3	3.19±0.14	-	-		-	-	-	-	-	-	-	
1,3-Dithiolo[4,5-b][1,3]dithiolo[4,5-E]pyridine-2,6-dione, 8-(trifluoromethyl)-	sulfur	SUL4	-	2.6±0.54	-	-	-	-	-	-	-	-	-	-
2-(3-Phenylpropyl-2-enylidene-hydrazono)-3-methyl-2,3-dihydrobenzothiazole	sulfur	SUL5	-	-	-	-	-	-	-	-	5.61±0.54	-	-	-
3-(3-Methylthiophen-2-yl)-6-(thiophen-	sulfur	SUL6	-	-	-	16.14±1.42	-	-	-	-	-	-	-	-

3-yl)-[1,2,4]triazolo[3,4-b][1,3,4]thiadiazole														
Diethyl Phthalate	sulfur	SUL7	-	-	-	-	-	-	-	-	-	4.39±0.21	-	-
Thiophene, tetrahydro-, 1,1-dioxide	sulfur	SUL8	-	-	28.54±1.52	-	-	-	-	2.18±0.02	-	-	-	-

NB: “-” indicates not detected

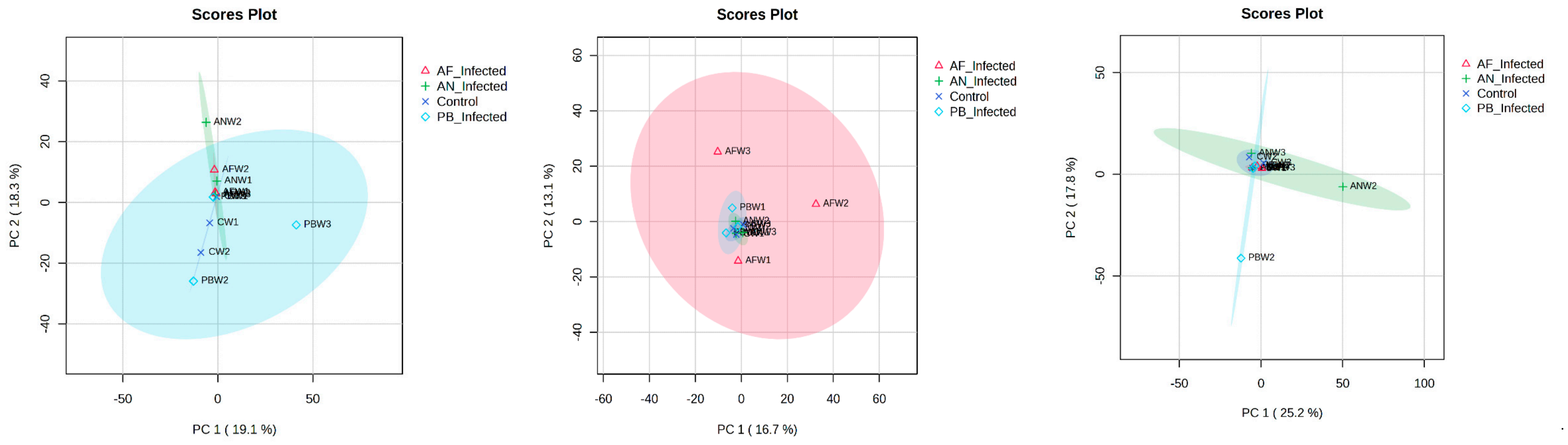

(A) **(B)** **(C)**

Figure S1: PCA plots of the VOCs recorded from different potato samples stored at (A) 25 °C (B) 8 °C and (C) 4 °C
